# Clinical Experience With Ivermectin and Nitazoxanide in the Management of COVID-19 Among Mexican Out- and Inpatients

**DOI:** 10.7759/cureus.74513

**Published:** 2024-11-26

**Authors:** Jorge O García-Méndez, Luis E Fernández-Garza, Karen Vallejo-Oviedo, Diana I Gómez-Curiel, Silvia A Barrera-Barrera, Rosario Ordaz-Cuellar, Jesús O Sosa-García, Rogelio A García-Torrentera, Eduardo Cervera, Hugo A Barrera-Saldaña

**Affiliations:** 1 Internal Medicine, Instituto Nacional de Cancerología, Mexico City, MEX; 2 Internal Medicine, General Hospital of Zone No. 2, Mexican Institute of Social Security, Monterrey, MEX; 3 Biotechnology, Laboratorio Nacional LANSEIDI, Monterrey, MEX; 4 Hematology, Instituto Nacional de Cancerología, Mexico City, MEX; 5 Biotechnology, Laboratorio Nacional LANSEIDI, Mexico City, MEX

**Keywords:** covid-19, covid-19 management, ivermectin, nitazoxanide, sars-cov-2

## Abstract

Background and objective

The use of ivermectin and nitazoxanide in the treatment of coronavirus disease 2019 (COVID-19) has been a subject of controversy. In this study, we aimed to describe our clinical experience in treating COVID-19 patients with these drugs in Mexico.

Material and methods

The study involved out- and inpatient clinical assessments of COVID-19 patients conducted in Mexico City from September 2020 to November 2021. Outpatients were treated with either ivermectin, nitazoxanide, or both drugs, while all inpatients received both. Clinical and laboratory analyses were used to assess the results.

Results

Of the 228 subjects in the outpatient group, 26.8% received ivermectin, 25.4% nitazoxanide, and 47.8% both. The proportion of negative polymerase chain reaction (PCR) was highest in patients treated late with ivermectin (≥5 days after symptom onset; p=0.004), followed by those receiving late treatment with nitazoxanide, and those with the combination at any time. The inpatient group had 179 subjects. A significant increase was seen in neutrophil, lymphocyte, monocyte, ferritin, and D-dimer levels, while an opposite trend was observed for C-reactive protein (CRP) and fibrinogen levels. Mechanical ventilation requirement was 15.5%, and 5% died during hospitalization.

Conclusions

Despite the limitations of our study, based on its findings, ivermectin and nitazoxanide could be useful in reducing the viral load, the requirement for mechanical ventilation, proinflammatory and procoagulant parameters, and the fatality rate in COVID-19 patients. Controlled clinical trials evaluating this combination should be carried out to determine its true usefulness and safety profile.

## Introduction

In December 2019, an outbreak of a novel coronavirus referred to as severe acute respiratory syndrome coronavirus 2 (SARS-CoV-2) was reported in China. The WHO termed the illness caused by SARS-CoV-2 as coronavirus disease 2019 (COVID-19) [1]. After the first cases were reported, COVID-19 rapidly spread throughout the world; on March 11, 2020, the WHO declared this disease a global pandemic [2]. Throughout the pandemic, several studies were carried out about the treatment of COVID-19. Studies have documented SARS-CoV-2 affecting various organs, leading to a wide range of symptoms among those affected by COVID-19. Although most COVID-19 cases have mild or moderate courses, up to 5-10% can have severe, potentially life-threatening courses, emphasizing the urgent need for cost-effective treatments [3].

Two of the drugs used against COVID-19 during the pandemic were ivermectin and nitazoxanide. Ivermectin has proven antiparasitic, antibacterial, and antiviral activity. The in vitro antiviral activity of ivermectin has been proven against a wide variety of DNA and RNA viruses [4]. Nevertheless, its in vivo antiviral capacity only has been proven against the Newcastle disease virus, the West Nile virus, the pseudorabies virus, and parvoviruses [4]. Nitazoxanide has been shown to have antiparasitic and antiviral activity [5]. In addition, it has been demonstrated to have a modulating effect on the inflammatory response, by suppressing the levels of proinflammatory cytokines such as tumor necrosis factor alpha (TNF-α) and interleukin-6 (IL-6) [5]. While exploring treatment options at the beginning of the pandemic, it emerged that both drugs had in vitro activity against the replication of SARS-CoV-2 [6,7].

However, five years after the start of clinical trials involving these drugs, there is still controversy about their usefulness. Meta-analyses examining both drugs have revealed contradictory results. Regarding ivermectin, while some have not shown any usefulness, others have shown a decrease in mortality in general, with even better results for severe COVID-19 and the drug's early clinical use [8-14]. Also, others have shown benefits in terms of the need for mechanical ventilation, in reverse transcription coupled with quantitative polymerase chain reaction (RT-qPCR) test results, and improvement in symptom relief [15,16]. As for nitazoxanide, a less studied drug, improvement has been observed in terms of decreasing viral load, decreasing supplemental oxygen requirements, as well as favorable results in some biochemical studies such as leukocyte levels, lactate dehydrogenase (LDH), and D-dimer [17-19]. This study aims to describe the clinical features and outcomes in COVID-19 patients treated with ivermectin and/or nitazoxanide at different levels of medical care in Mexico City during the early stages of the pandemic.

## Materials and methods

We conducted a prospective and descriptive study of consecutive patients with a diagnosis of COVID-19 [20]. The diagnosis was confirmed by a government-recognized COVID-19 diagnostic laboratory (Columbia Biotec, Mexico City, Mexico) [21]. The study involved both out- and inpatient clinical experiences with ivermectin and nitazoxanide in the early stages of the pandemic, a period characterized by its overwhelming progress, the emergency scenarios that made clinical practitioners deal with it according to their best judgment, and a lack of standardized effective treatment.

The clinical protocol was approved by the Research and Ethics Committee of the National Cancer Institute (registration code: NTIVCOL-2020-01). The study was carried out under the guidelines established in the statement of the national health regulatory agency (Federal Commission for Protection Against Sanitary Risks, COFEPRIS) on the use of drugs of unproven efficacy in COVID-19 patients, and a signed informed consent was obtained in all cases. Due to the health emergency prevailing at that time and the few studies published on the subject at the time, we did not engage in the calculation of a specific sample size, and all patients who met the inclusion criteria for the study were included: patients of legal age, of both sexes, with a positive test for COVID-19.

**Figure 1:**
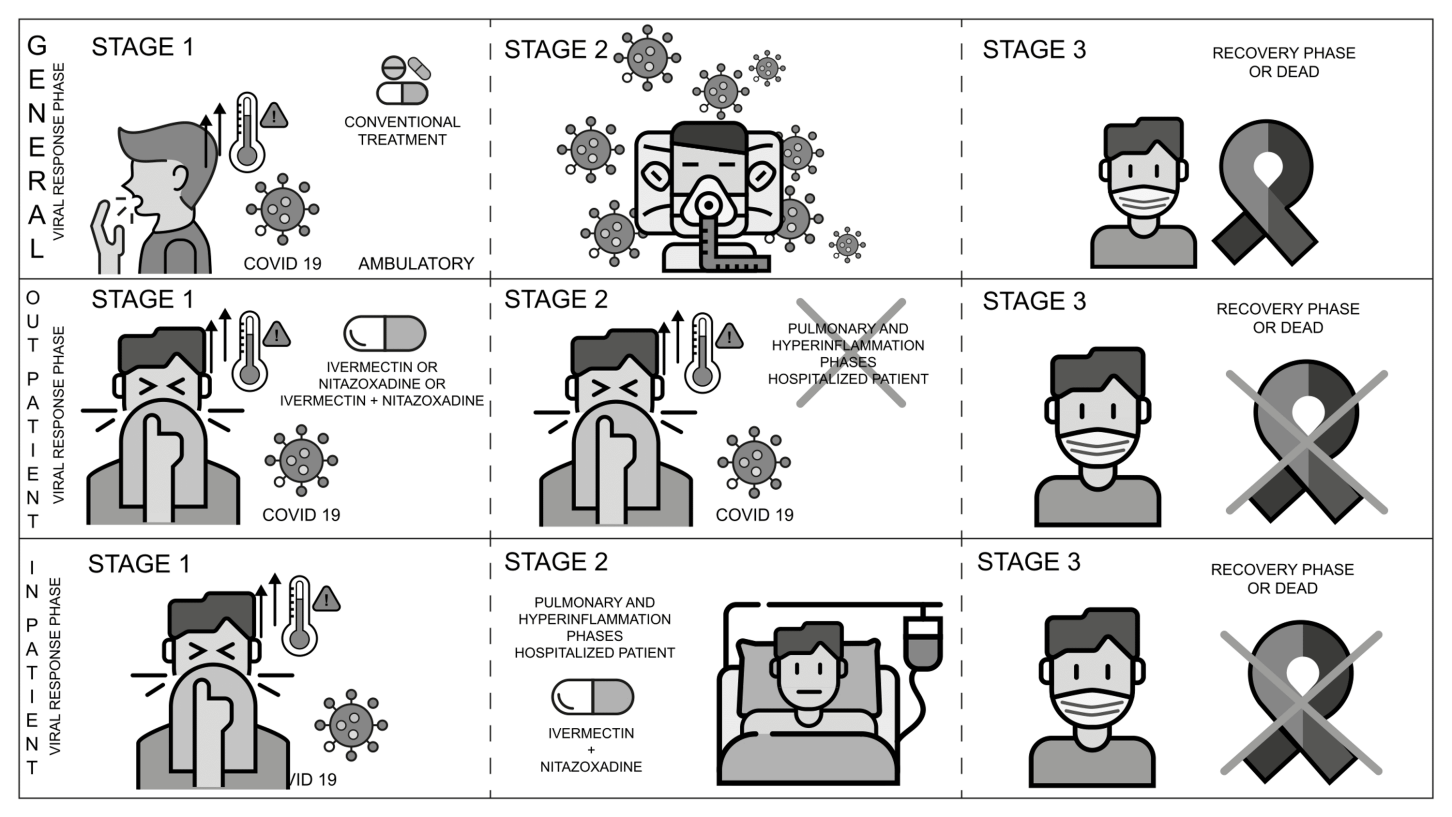
Design of the therapeutic interventions for the repurposing of ivermectin and nitazoxanide in the treatment of COVID-19 infection The research consisted of the clinical experience of treating two types of patients: out- and inpatients COVID-19: coronavirus disease 2019

The clinical assessment with outpatients was conducted in a private hospital in Mexico City (Medica Sur clinic) from September 2020 to September 2021, and they were divided into three treatment groups: ivermectin, nitazoxanide, and the combination of both (dosage - ivermectin: 4 mg every eight hours for five days; nitazoxanide: 500 mg also every eight hours for five days) (Figure 1). For the analyses of outcomes, interventions were classified according to the time of evolution of the clinical picture, to investigate in which phase of the infection it could be more useful to implement the treatment. The clinical analysis of hospitalized patients was held at the same private hospital from November 2020 to October 2021, with all the patients being considered at high risk for disease progression to critical stages and having been prescribed the combination of both drugs (same posology of outpatients) together with the standard treatment (steroids and anticoagulants).

SARS-CoV-2 nucleic acid testing was carried out on naso and oropharyngeal swabs. The samples were transported in a universal viral transportation medium prepared as per the recipe of the Centers for Disease Control and Prevention (CDC). Nucleic acid extraction was performed using RNA extraction kits (commercial manual or automatic). RT-qPCR was carried out on the extracted RNA [22]. The Ct-value was considered a surrogate of viral load and those laboratory results rendering Ct values ≤37 were considered positive [23]. Clinical and epidemiological data were extracted using a standardized medical history form. Outpatients underwent daily monitoring of their symptoms and adherence to treatment by telephone and were scheduled to carry out their PCRs. As for inpatients, laboratory studies [hemogram, C-reactive protein (CRP), ferritin, D-dimer, and fibrinogen] were carried out upon admission and were repeated as required during their hospital stay.

Continuous variables were summarized as the median and interquartile range (IQR) or the mean ± standard deviation (SD), and categorical variables as percentages. The normality of distribution was analyzed with the Kolmogorov-Smirnov test. The patients were divided into groups according to the treatment received - ivermectin or nitazoxanide or both - and according to the time from the onset of the first symptom to when these treatments were started: <5 (early) and ≥5 (late) days. The differences between groups were tested using a one-way ANOVA test for variables from more than two groups normally distributed and unrelated; Mann-Whitney U test for variables from two groups non-normally distributed and unrelated; Friedman test for variables from more than two groups non-normally distributed and related; and Kruskal-Wallis test for variables from more than two groups non-normally distributed and unrelated. The difference between categorical variables was determined using the Chi-square test. A p-value <0.05 was considered statistically significant. Statistical analyses were performed using SPSS Statistics v22 (IBM Corp., Armonk, NY) and RStudio v1.4.1717.

## Results

The study included 228 outpatients, with a median age of 42 (31-52) years; 125 (54.8%) were female. The mean BMI was 26.61 ± 4.5 kg/m², with four (1.8%) underweight, 85 (37.3%) overweight, and 48 (21.1%) obese patients. The most frequent comorbidities were arterial hypertension in 30 (13.2%), type 2 diabetes (T2D) in 20 (8.8%), and asthma in 10 (4.4%). Regarding previous medication, 54 (23.7%) patients had received at least one drug. The most frequent groups of medication were antihypertensive (n=26, 11.4%) and antidiabetics (n=15, 6.6%) (Table 1).

**Table 1:** Baseline characteristics of COVID-19 out- and inpatients ACE: angiotensin-converting enzyme; ARBs: angiotensin II receptor blockers; CCB: calcium channel blockers; CKD: chronic kidney disease; COPD: chronic obstructive pulmonary disease; COVID-19: coronavirus disease 2019; DPP-4: dipeptidyl peptidase 4; NA: not available; IQR: interquartile range; SD: standard deviation; SGLT2: sodium-glucose cotransporter-2; T2D: type 2 diabetes

Characteristics	Outpatients (n=228)	Inpatients (n=179)
Age, years, median (IQR), or mean ± SD	42 (31-52)	52.2 ± 14.3
Sex, female, n (%)	125 (54.8)	133 (74.3)
Comorbidities, n (%)		
Arterial hypertension	30 (13.2)	41 (22.9)
T2D	20 (8.8)	26 (14.5)
Asthma	10 (4.4)	NA
Cardiovascular disease	NA	15 (8.4)
History of cancer	NA	11 (6.1)
COPD	NA	2 (1.1)
CKD	NA	2 (1.1)
Previous medication, n (%)		
Antihypertensive	26 (11.4)	34 (19)
ARBs	17 (7.4)	24 (13.4)
CCB	9 (3.9)	3 (1.7)
ACE inhibitors	4 (1.7)	2 (1.1)
Beta-blockers	3 (1.3)	11 (6.1)
Antidiabetics	15 (6.6)	26 (14.5)
Biguanides	13 (5.7)	24 (13.4)
Sulfonylureas	3 (1.3)	3 (1.7)
Thiazolidinediones	1 (0.4)	0
DPP-4 inhibitors	1 (0.4)	6 (3.5)
Insulin	1 (0.4)	4 (2.2)
SGLT2 inhibitors	0	1 (0.6)
Lipid-lowering agents	4 (1.8)	12 (6.7)
Statins	3 (1.3)	11 (6.1)
Fibrates	2 (0.9)	2 (1.1)
Ezetimibe	1 (0.6)	1 (0.6)
Antiplatelet agents	0	7 (3.9)
Acetylsalicylic acid	0	6 (3.4)
Clopidogrel	0	1 (0.6)
Levothyroxine	6 (2.6)	9 (5)
Benzodiazepines	5 (2.2)	1 (0.6)
Antidepressants	5 (2.2)	1 (0.6)
Antiepileptics	5 (2.2)	0
Bronchodilators	0	4 (2.2)
Diuretics	4 (1.7)	2 (1.1)
Antineoplastic agents	0	3 (1.7)
Antiprostatic hypertrophy agents	1 (0.4)	3 (1.7)

During the clinical evaluation, the median duration of symptoms was six (four to eight) days. The most frequent symptoms were headache (n=142, 62.3%), cough (n=133, 58.3%), and fatigue (n=99, 43.4%). Of note, 171 (75%) patients had already taken some medication for the current disease, and the most frequent medications were acetaminophen (n=106, 46.5%), ibuprofen (n=37, 16.2%), and azithromycin (n=25, 11%). Twenty-six (11.4%) patients required supplemental oxygen and seven (3.1%) hospitalization; 41 (18%) patients were lost to follow-up. Of the remaining 187 patients, 64 (34.2%) showed negative PCR by the first week, and 128 (68.4%) by the end of the second week (Table 2).

**Table 2:** Clinical evaluation of COVID-19 outpatients COVID-19: coronavirus disease 2019; IQR: interquartile range; SD: standard deviation

Variables	Values (n=228)
Duration of symptoms, days, median (IQR)	6 (4-8)
Headache, n (%)	142 (62.3)
Duration of headache in days, median (IQR)	4 (2-5)
Cough, n (%)	133 (58.3)
Duration of cough in days, median (IQR)	5 (3-8)
Fatigue, n (%)	99 (43.4)
Duration of fatigue in days, median (IQR)	3 (2-5)
Abdominal pain, n (%)	89 (39)
Duration of abdominal pain in days, median (IQR)	2 (1-4)
Anosmia, n (%)	68 (29.8)
Duration of anosmia in days, median (IQR)	5 (4-6)
Diarrhea, n (%)	65 (28.5)
Duration of diarrhea in days, median (IQR)	2 (1-3)
Myalgias, n (%)	58 (25.4)
Duration of myalgias in days, median (IQR)	2 (2-4)
Fever, n (%)	41 (18)
Duration of fever in days, median (IQR)	2 (1.5-4)
Dyspnea, n (%)	26 (11.4)
Duration of dyspnea in days, median (IQR)	4 (2-5)
Arthralgias, n (%)	21 (9.2)
Duration of arthralgia in days, median (IQR)	3 (1-5)
Physical examination	
Weight, kg, mean ± SD	73.43 ± 16.08
Height, m, mean ± SD	1.65 ± 0.09
BMI, kg/m^2^, mean ± SD	26.61 ± 4.5
Oxygen saturation at 1º day, %, median (IQR)	94 (92-95.25)
Supplemental oxygen requirement, n (%)	26 (11.4)
Hospitalization, n (%)	7 (3.1)
Medication use before the first evaluation, n (%)	171 (75)
Acetaminophen, n (%)	106 (46.5)
Ibuprofen, n (%)	37 (16.2)
Azithromycin, n (%)	25 (11)
Acetylsalicylic acid, n (%)	24 (10.5)
Ivermectin, n (%)	22 (9.6)
Chlorphenamine, n (%)	18 (7.9)
Vitamin C, n (%)	17 (7.5)
Dexamethasone, n (%)	14 (6.1)
Cholecalciferol, n (%)	13 (5.7)
Amantadine, n (%)	12 (5.3)
Apixaban, n (%)	10 (4.4)
Loratadine, n (%)	10 (4.4)
Phenylephrine, n (%)	9 (3.9)
Amoxicillin, n (%)	9 (3.9)
Metamizole, n (%)	8 (3.5)
Omeprazole, n (%)	8 (3.5)
Ceftriaxone, n (%)	8 (3.5)
Ambroxol, n (%)	8 (3.5)
Dextromethorphan, n (%)	7 (3.1)
Oseltamivir, n (%)	7 (3.1)
Clavulanic acid, n (%)	6 (2.6)
Dropropizine, n (%)	6 (2.6)
Naproxen, n (%)	5 (2.2)
Diclofenac, n (%)	5 (2.2)
Prednisone, n (%)	5 (2.2)
Ciprofloxacin, n (%)	5 (2.2)
Clarithromycin, n (%)	5 (2.2)
Laboratory findings – follow-up, n (%)	
First week (n=184)	
Negative	64 (34.8)
Low viral load	96 (52.2)
Medium viral load	18 (9.8)
High viral load	6 (3.3)
Second week (n=187)	
Negative	128 (68.4)
Low viral load	57 (30.5)
Medium viral load	2 (1.1)

**Figure 2:**
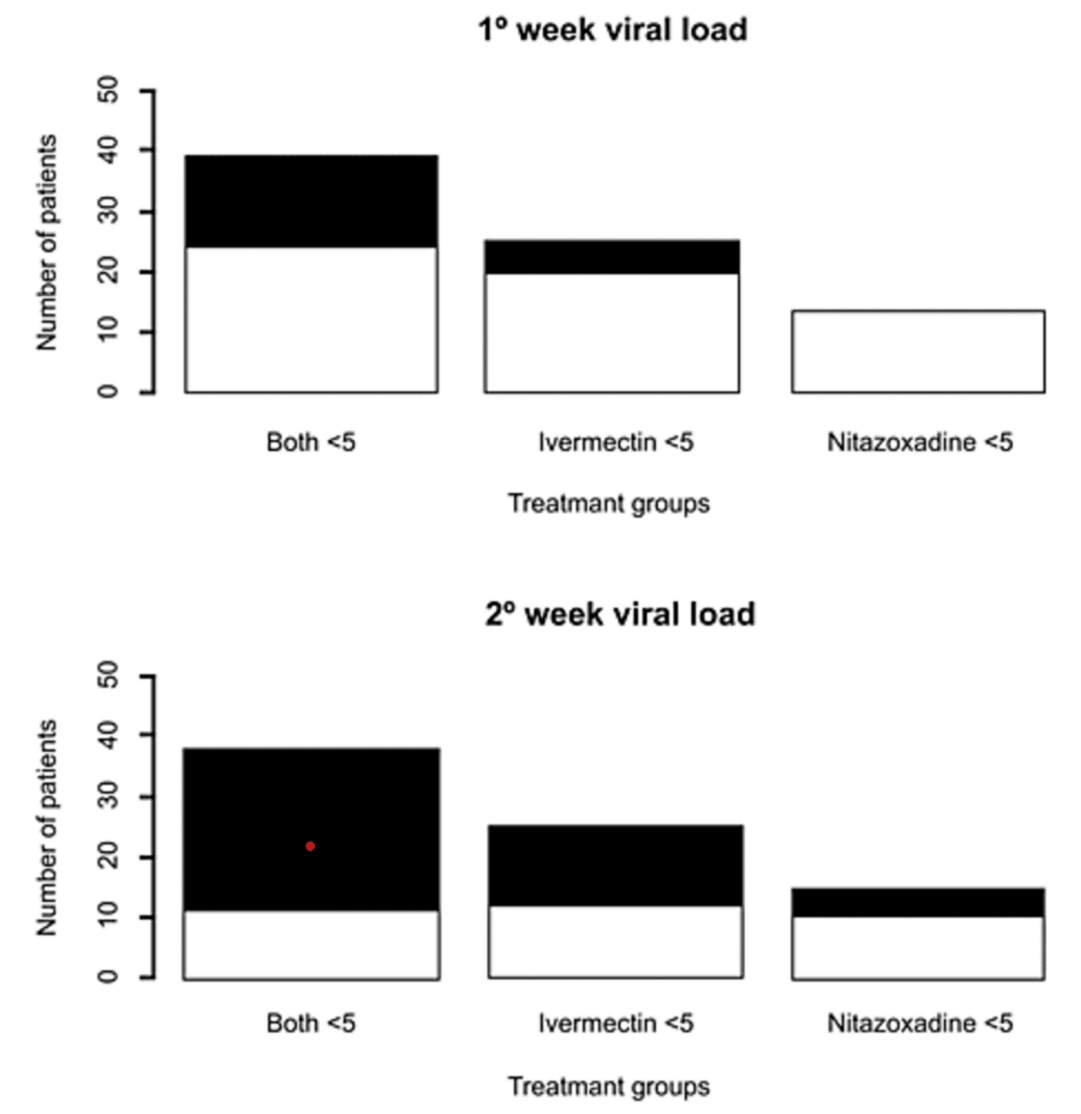
Comparison of viral load at one and two weeks after starting different types of treatments in COVID-19 outpatients Black: undetectable. White: detectable COVID-19: coronavirus disease 2019

Regarding the treatment groups, 61 (26.8%) received ivermectin, 58 (25.4%) nitazoxanide, and 109 (47.8%) both. When comparing the groups according to the treatment they received and the number of days after the beginning of the clinical picture they started the treatment (ivermectin <5 vs. ivermectin ≥5 vs. nitazoxanide <5 vs. nitazoxanide ≥5 vs. both <5 vs. both ≥5), we observed that the proportions of patients with negative PCR at the first and second week after starting the treatments were higher among those treated with ivermectin ≥5 days after the onset of symptoms (first week: 21.4% vs. 50% vs. 0% vs. 33.3% vs. 34.1% vs. 26.5%, p=0.013; second week: 46.4% vs. 86.6% vs. 31.2% vs. 64.1% vs. 61.3% vs. 61.2%, p=0.004) (Figure 2).

**Figure 3:**
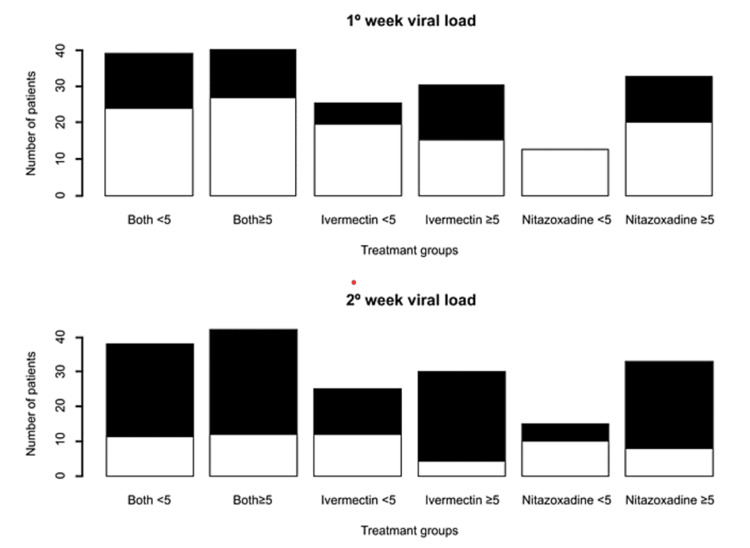
Comparison of viral load at one and two weeks after starting different types of treatments in outpatients with early COVID-19 Black: undetectable. White: detectable COVID-19: coronavirus disease 2019

Also, when comparing only the three groups with <5 days, we found that the group with both drugs had a greater proportion of negative PCR tests during one (21.4% vs. 0% vs. 34.1%, p=0.021) and two weeks (46.4% vs. 31.2% vs. 61.3%, p=0.099) after initiating treatments (Figure 3). Other statistically significant differences between the six groups were seen in terms of age (39.2 vs. 46 vs. 43.8. vs. 37.6 vs. 46.4 vs. 43.1 years, p=0.038), history of arterial hypertension (7.1% vs. 16.6% vs. 12.5% vs. 2.5% vs. 27.2% vs. 12.2%, p=0.029), and the use of ivermectin before consultation at our institution (17.8% vs. 33.3% vs. 0% vs. 10.2% vs. 2.2% vs. 4.1%, p<0.001] (Table 3).

**Table 3:** Comparison between treatment groups of COVID-19 outpatients ^*^Statistically significant AH: arterial hypertension; BMI: body mass index; IQR: interquartile range; SD: standard deviation; T2D: type 2 diabetes mellitus; VL: viral load

Variable or condition	+Ivermectin <5 d (n=28)	+Ivermectin ≥5 d (n=30)	+Nitazoxanide <5 d (n=16)	+Nitazoxanide ≥5 d (n=39)	+Both <5 d (n=44)	+Both ≥5 d (n=4)	P-value
Age, years, mean ± SD	39.29 ± 13.26	46 ± 13.52	43.81 ± 16.44	37.68 ± 12.48	46.48 ± 13.7	43.1 ± 13.99	0.038^*^
Sex, female, n (%)	17 (60.7)	16 (53.3)	9 (56.3)	24 (61.5)	16 (36.4)	30 (54.4)	0.157
BMI, kg/m^2^, mean ± SD	27.63 ± 4.86	27 ± 3.66	25.7 ± 2.88	25.37 ± 4.38	27.07 ± 5.08	26.62 ± 5.14	0.366
AH, n (%)	2 (7.1)	5 (16.6)	2 (12.5)	1 (2.5)	12 (27.2)	6 (12.2)	0.029^*^
T2D, n (%)	2 (7.1)	0	1 (6.2)	3 (7.6)	6 (13.6)	7 (14.2)	0.306
Prior acetaminophen, n (%)	13 (46.4)	17 (56.6)	8 (50)	18 (46.1)	22 (50)	26 (53.1)	0.956
Prior ibuprofen, n (%)	4 (14.2)	6 (20)	2 (12.5)	10 (25.6)	2 (4.5)	13 (26.5)	0.074
Prior azithromycin, n (%)	3 (10.7)	5 (16.6)	0	6 (15.3)	4 (9.1)	7 (14.2)	0.575
Prior acetylsalicylic acid, n (%)	2 (7.1)	1 (3.3)	2 (12.5)	8 (20.5)	4 (9.1)	7 (14.2)	0.290
Prior ivermectin, n (%)	5 (17.8)	10 (33.3)	0	4 (10.2)	1 (2.2)	2 (4.1)	<0.001^*^
Duration of symptoms, days, median (IQR)	5 (3-8)	5 (4-7)	6 (4-8)	6 (5-8)	6 (5-7)	6 (4-8)	0.755
Headache, n (%)	16 (57.1)	22 (73.3)	7 (43.8)	23 (59)	29 (65.9)	36 (73.5)	0.222
Cough, n (%)	19 (67.9)	20 (66.7)	7 (43.8)	28 (71.8)	23 (52.3)	32 (65.3)	0.257
Fatigue, n (%)	13 (46.4)	12 (40)	7 (43.8)	21 (53.8)	23 (52.3)	6 (12.2)	<0.001
Abdominal pain, n (%)	9 (32.1)	13 (43.3)	10 (62.5)	17 (43.6)	20 (45.5)	19 (38.8)	0.506
Diarrhea, n (%)	6 (21.4)	8 (26.7)	5 (31.3)	16 (41)	14 (31.8)	16 (32.7)	0.652
Myalgias, n (%)	8 (28.6)	10 (33.3)	6 (37.5)	10 (25.6)	10 (22.7)	14 (28.6)	0.868
Anosmia, n (%)	8 (28.6)	8 (26.7)	7 (43.8)	12 (30.8)	15 (34.1)	17 (34.7)	0.876
Fever, n (%)	1 (3.6)	6 (20)	2 (12.5)	8 (20.5)	10 (22.7)	22 (44.9)	0.001^*^
Arthralgias, n (%)	1 (3.6)	6 (20)	2 (12.5)	6 (15.4)	2 (4.5)	3 (6.1)	0.132
Dyspnea, n (%)	3 (10.7)	3 (10)	2 (12.5)	5 (12.8)	2 (4.5)	8 (16.3)	0.632
Oxygen requirement, n (%)	3 (10.7)	0	3 (18.7)	2 (5.1)	5 (11.3)	9 (18.3)	0.109
Hospitalization, n (%)	1 (3.5)	0	0	1 (2.5)	2 (4.5)	2 (4.1)	0.836
1º week VL negative (n=180). n (%)	6 (21.4)	15 (50)	0	13 (33.3)	15 (34.1)	13 (26.5)	0.013^*^
2º week VL negative (n=183), n (%)	13 (46.4)	26 (86.6)	5 (31.2)	25 (64.1)	27 (61.3)	30 (61.2)	0.004^*^

We included 179 hospitalized patients, with a mean age of 52.2 ± 14.3 years; 133 (74.3%) were male. The mean BMI was 27.89 ± 4.46 kg/m², with 73 (40.8%) being overweight and 55 (30.9%) obese. The most frequent comorbidities were arterial hypertension (n=41, 22.9%), T2D (n=26, 14.5%), and cardiovascular disease (n=15, 8.4%). The most frequent previously used groups of medications were antihypertensives (n=34, 19%), antidiabetics (n=26, 14.5%), and lipid-lowering agents (n=12, 6.7%) (Table 1).

The mean time from initiation of symptoms to hospital admission was 8.44 ± 4.57 days. The most common symptoms were fever (n=174, 97.2%), cough (n=164, 91.6%), and dyspnea (n=163, 91.1%). Regarding the evaluation in the emergency room, 69 (38.5%) had hyperthermia, 78 (43.6%) arrived with tachycardia, 83 (46.4%) of the patients had tachypnea, 96 (53.6%) were hypoxemic, 46 (25.7%) of patients arrived with hypertensive values, while only 15 (8.4%) showed up with hypotension (Table 4).

**Table 4:** Initial assessment of COVID-19 inpatients in the emergency department COVID-19: coronavirus disease 2019; BMI: body mass index; CRP: C-reactive protein; IQR: interquartile range; SD: standard deviation

Variables	Values (n=179)
Days from symptoms onset to hospital admission, mean ± SD	8.44 ± 4.57
Fever, n (%)	174 (97.2)
Cough, n (%)	164 (91.6)
Dyspnea, n (%)	163 (91.1)
Headache, n (%)	160 (89.4)
Shaking chills, n (%)	150 (83.8)
Abdominal pain, n (%)	147 (82.1)
Diarrhea, n (%)	134 (74.9)
Sore throat, n (%)	124 (69.3)
Anosmia, n (%)	113 (63.1)
Ageusia, n (%)	82 (45.8)
Malaise, n (%)	18 (10.1)
Fatigue, n (%)	15 (8.4)
Myalgia, n (%)	11 (6.1)
Arthralgia, n (%)	10 (5.6)
Rhinorrhea, n (%)	5 (2.8)
Epiphora, n (%)	2 (1.1)
Chest pain, n (%)	2 (1.1)
Bradypsychia, n (%)	2 (1.1)
Bradylalia, n (%)	1 (0.6)
Hyporexia, n (%)	1 (0.6)
Genital pain, n (%)	1 (0.6)
Physical examination	
Weight, kg, mean ± SD	1.7 (1.62-1.74)
Height, m, mean ± SD	1.68 ± 0.09
BMI, kg/m^2^, mean ± SD	27.89 ± 4.46
Body temperature, ºC, median (IQR)	37.2 (36.7-38)
Heart rate, bpm, mean ± SD	98.14 ± 15.57
Respiratory rate, rpm, median (IQR)	20 (20-25)
Oxygen saturation, %, median (IQR)	88 (84.75-92.25)
Systolic BP, mmHg, mean ± SD	127.36 ± 18.15
Diastolic BP, mmHg, mean ± SD	74.02 ± 11.81
Mean BP, mmHg, mean ± SD	91.8 ± 12.39
Medication use before hospitalization	
Number of medications, median (IQR)	2 (1-4)
Acetaminophen, n (%)	84 (46.9)
Azithromycin, n (%)	49 (27.4)
Ivermectin, n (%)	49 (27.4)
Dexamethasone, n (%)	32 (17.9)
Ceftriaxone, n (%)	17 (9.5)
Enoxaparin, n (%)	16 (8.9)
Nitazoxanide, n (%)	15 (8.4)
Oseltamivir, n (%)	13 (7.3)
Ibuprofen, n (%)	11 (6.1)
Clarithromycin, n (%)	10 (5.6)
Acetylsalicylic acid, n (%)	9 (5)
Prednisone, n (%)	8 (4.5)
Levofloxacin, n (%)	8 (4.5)
Rivaroxaban, n (%)	6 (3.4)
Apixaban, n (%)	6 (3.4)
Hydroxychloroquine, n (%)	4 (2.2)
Amoxicillin, n (%)	4 (2.2)
Clavulanic acid, n (%)	4 (2.2)
Cholecalciferol, n (%)	4 (2.2)
Vitamin C, n (%)	4 (2.2)
Salbutamol, n (%)	4 (2.2)
Metamizole, n (%)	4 (2.2)
Laboratory findings, median (IQR)	
WBC count, x 10^9^/L	7.6 (5.37-10.8)
Neutrophil count, x 10^9^/L	5.79 (4.05-9.38)
Lymphocyte count, x 10^9^/L	0.74 (0.53-1.09)
Monocyte count, x 10^9^/L	0.41 (0.28-0.62)
Ferritin, mg/L	542.5 (286-949.9)
CRP, mg/L	104 (44.38-165.48)
D-dimer, ng/mL	625 (432.5-985)
Fibrinogen, mg/dL	540 (421-676)

The median number of medications used for the current illness before arrival at the emergency room was two (one to four). The most frequent drugs used were acetaminophen (n=84, 46.9%), azithromycin (n=49, 27.4%), and ivermectin (n=49, 27.4%). Regarding the laboratory results, 44 (24.6%) patients had leukocytosis, 11 (6.1%) leukopenia, 59 (33%) patients had neutrophilia, three (1.7%) neutropenia, 113 (63.1%) patients had lymphopenia, 22 (12.3%) patients had monocytosis, and 14 (7.8%) had monocytopenia. Of note, 131 (73.2%) patients had a value above normal for ferritin, 168 (93.9%) patients had values above the upper limit of normal for CRP, and 106 (59.2%) patients had values above normal for D-dimer (Table 4).

During hospitalization, 172 (96.1%) patients required supplemental oxygen, and 28 (15.6%) patients required the use of mechanical ventilation. All received ivermectin and nitazoxanide; 173 (96.6%) received enoxaparin, 116 (64.8%) methylprednisolone, and 101 (56.4%) received acetaminophen. The median duration of hospital stay was nine (7-14) days and nine (5%) patients died during hospitalization (Table 5).

**Table 5:** Treatment during hospitalization of COVID-19 inpatients COVID-19: coronavirus disease 2019; IQR: interquartile range; SD: standard deviation

Variables	Values (n=179)
Supplemental oxygen requirement, n (%)	179 (100)
Days with supplemental oxygen, median (IQR)	9 (7-14)
Mechanical ventilation requirement, n (%)	28 (15.6)
Days with mechanical ventilation, mean ± SD	19.11 ± 12.62
Ivermectin, n (%)	179 (100)
Nitazoxanide, n (%)	179 (100)
Enoxaparin, n (%)	173 (96.6)
Methylprednisolone, n (%)	116 (64.8)
Acetaminophen, n (%)	101 (56.4)
Dexamethasone, n (%)	30 (16.8)
Ruxolitinib, n (%)	26 (14.5)
Tocilizumab, n (%)	16 (8.9)
Apixaban, n (%)	7 (3.9)
Azithromycin, n (%)	6 (3.4)
Ceftriaxone, n (%)	6 (3.4)
Prednisone, n (%)	4 (2.2)
Hydroxychloroquine, n (%)	2 (1.1)
Acetylcysteine, n (%)	2 (1.1)
Acyclovir, n (%)	2 (1.1)
Baricitinib, n (%)	2 (1.1)
Linezolid, n (%)	2 (1.1)
Clarithromycin, n (%)	1 (0.6)
Clindamycin, n (%)	1 (0.6)
Acalabrutinib, n (%)	1 (0.6)
Vancomycin, n (%)	1 (0.6)
Vortioxetine, n (%)	1 (0.6)
Acetylsalicylic acid, n (%)	1 (0.6)
Piperacillin, n (%)	1 (0.6)
Tazobactam, n (%)	1 (0.6)
Rivaroxaban, n (%)	1 (0.6)
Dropropizine, n (%)	1 (0.6)
Ritonavir, n (%)	1 (0.6)
Lopinavir, n (%)	1 (0.6)
Length of hospital stay, days, median (IQR)	9 (7-14)
Death, n (%)	9 (5)

**Figure 4:**
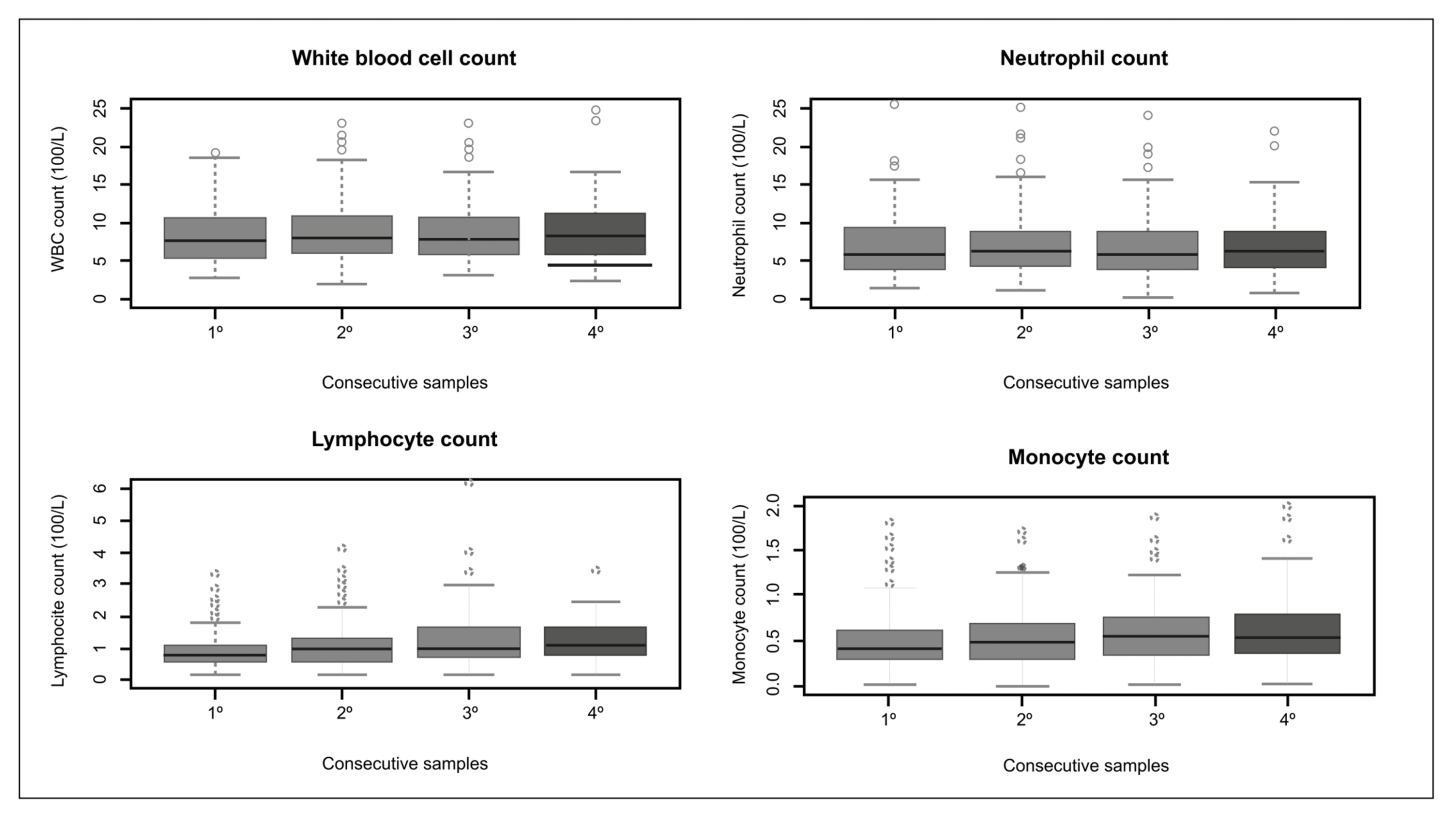
Differences among the results of four consecutive samples in hemogram parameters (white blood cell, neutrophil, lymphocyte, and monocyte counts)

**Figure 5:**
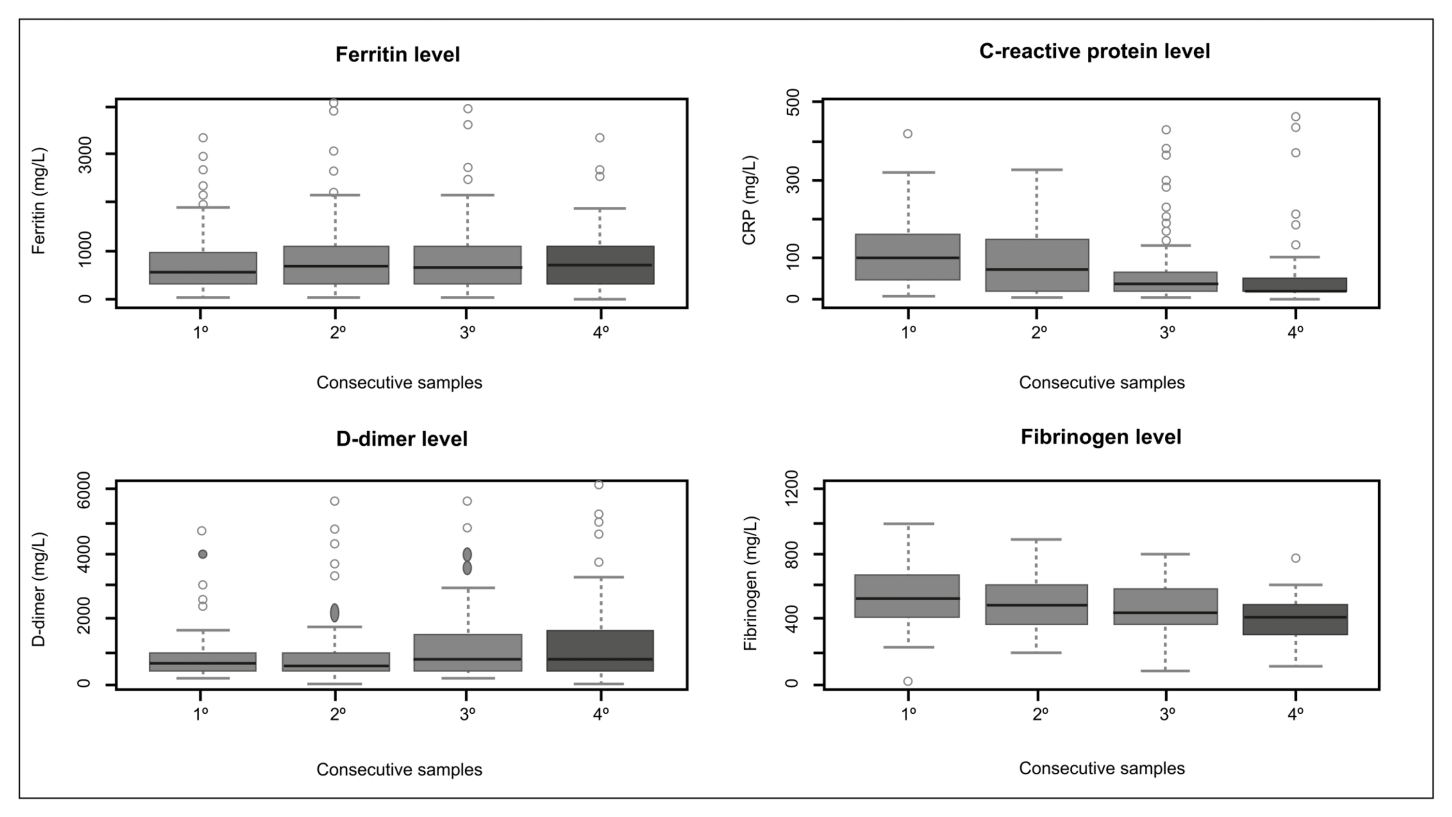
Differences among the results of four consecutive samples in the levels of ferritin, C-reactive protein, D-dimer, and fibrinogen

During the hospital stay, a comparison of the results of the most relevant laboratory findings was made involving four samples, which showed that most of the parameters increased after their first intake during admission, including the neutrophil count (5.79→6.24→6.08→6.16 x 10⁹/L, p=0.011), lymphocyte count (0.74→0.91→0.95→1.06 x 10⁹/L, p<0.001), monocyte count (0.4→0.47→0.51→0.53 x 10⁹/L, p=0.001), ferritin level (542.5→684.3→661.3→736 mg/L, p<0.001), D-dimer level (625→570→720→735 ng/mL, p=0.007). The only variables that showed a decrease were CRP level (104→65.61→30.43→15.24 mg/L, p<0.001) and fibrinogen level (540→494.5→435→415.5 mg/dL, p<0.001) (Table 6; Figures 4-5).

**Table 6:** Differences between laboratory study parameters during the hospital stay ^*^Statistically significant CRP: C-reactive protein; IQR: interquartile range

	1st sample, median (IQR)	2nd sample, median (IQR)	3rd sample, median (IQR)	4th sample, median (IQR)	P-value
WBC count, x 10^9^/L (n=170/160/118/77)	7.6 (5.37-10.8)	8 (6-10.9)	8.3 (5.9-10.72)	8.3 (5.85-11.25)	0.566
Neutrophil count, x 10^9^/L (n=170/159/118/76)	5.79 (4.05-9.38)	6.24 (4.22-9.11)	6.08 (4.06-9.01)	6.16 (3.94-9.09)	0.011^*^
Lymphocyte count, x 10^9^/L (n=170/160/118/76)	0.74 (0.53-1.09)	0.91 (0.57-1.31)	0.95 (0.67-1.68)	1.06 (0.81-1.63)	<0.001^*^
Monocyte count, x 10^9^/L (Nn170/160/118/77)	0.4 (0.28-0.62)	0.47 (0.29-0.68)	0.51 (0.34-0.77)	0.53 (0.37-0.79)	0.001^*^
Ferritin level, mg/L (n=167/149/117/73)	542.5 (286-949.9)	684.3 (326.85-1086.3)	661.3 (344.6-1146.75)	736 (319.3-1109.25)	<0.001^*^
CRP level, mg/L (n=171/151/121/78)	104 (44.38-165.48)	65.61 (18.37-154.75)	30.43 (6.3-62.84)	15.24 (3.78-53.91)	<0.001^*^
D-dimer level, ng/mL (n=168/152/117/78)	625 (432.5-985)	570 (392.5-947.5)	720 (395-1660)	735 (420-1612.5)	0.007^*^
Fibrinogen level, median (IQR), mg/dL (N= 139/112/89/48)	540 (421-676)	494.5 (372-615)	435 (352-579)	415.5 (311-491.5)	<0.001^*^

## Discussion

Ivermectin and nitazoxanide are affordable and safe drugs with antiviral properties, making them attractive options for treating COVID-19. Both drugs are part of the human pharmacopeia and are approved by COFEPRIS and the FDA for different indications. Ivermectin is used in the treatment of intestinal strongyloidiasis, onchocerciasis, head lice, and rosacea [24]. On the other hand, nitazoxanide is used in cryptosporidiosis and giardiasis [25]. The objective of our study is to describe the experience of treating patients confirmed with SARS-CoV-2 infection using these two drugs. The first group in the study consisted of patients who developed mild to moderate COVID-19 treated on an outpatient basis with the drugs separately or in combination. The second group comprised hospitalized patients with severe COVID-19 receiving the combination of both drugs. To our knowledge, this is the first study to show the effects of the combination of ivermectin and nitazoxanide alone; a previous study of both together with ribavirin and zinc reported a decrease in the time of detection of the virus by nasopharyngeal swab, similar to this study [26].

In the first group, we did not find any difference in the duration of symptoms, supplemental oxygen requirement, and hospitalization between the treatment schemes. Nevertheless, we found that a significantly higher proportion of patients treated late with ivermectin alone had negative RT-PCR tests both at the first (late ivermectin: 50% vs. early both: 34.1% vs. late nitazoxanide: 33.3% vs. late both: 26.5% vs. early ivermectin: 21.4% vs. early nitazoxanide: 0%, p=0.013) and second week (late ivermectin: 86.6% vs. late nitazoxanide: 64.1% vs. early both: 61.3% vs. late both: 61.2% vs. early ivermectin: 46.4% vs. early nitazoxanide: 31.2%, p=0.004) from the outset of the treatment. In a controlled clinical trial involving 30 patients, it was found that after seven days of treatment with ivermectin or placebo, 70% of the patients with undetectable viral load were in the ivermectin-treated group [27]. Also, a meta-analysis of 19 studies analyzing treatment with ivermectin showed a significantly higher negative RT-PCR test results rate (RR: 1.23, 95% CI: 1.01-1.51, p=0.04) and shorter time to negative RT-PCR test results (MD: -3.29, 95% CI: -5.69 - -0.89, p=0.007) [15].

It would be thought that the faster ivermectin therapy is implemented, the faster the viral load would decrease, as shown by Chaccour et al., who only included patients with less than 72 hours of the onset of symptoms. The group treated with ivermectin showed a tendency to have a faster decrease in the viral load and IgG titers; however, it was not statistically significant, probably due to the size of their sample (n=24) [28]. In contrast, the greatest benefit in our cohort was observed when the treatment started no later than day five of the onset of the symptoms. However, it should be noted that around 33.3% of the patients in that group had started to consume ivermectin before their first consultation.

Interestingly, by including only those patients treated early, we found that in the first week, the group receiving both drugs had the highest statistically significant proportion of negative samples (both: 34.1% vs. ivermectin: 21.5% vs. nitazoxanide: 0%, p=0.021). This could be explained by the findings of a study that compared viral load five days after starting treatment with nitazoxanide against placebo, where a statistically higher proportion of negative tests was found in the group treated with nitazoxanide, which suggests that nitazoxanide does add a beneficial factor to ivermectin treatment in patients treated promptly [29]. The latter has already been shown in two meta-analyses, which reported that nitazoxanide reduces the viral load in a statistically significant manner compared to placebo [17,18]. It is worth emphasizing that a decrease in viral load may be reflected in COVID-19 patients as a lesser severity of the disease, a lower rate of requiring hospitalization, and a decrease in the probability of spreading the virus [30].

Regarding findings in the second group, four study variables were considered: laboratory results, a requirement for mechanical ventilation, length of hospital stay, and fatality rate. The severity of COVID-19 can be characterized by a marked lymphopenia that affects both CD4+ and CD8+ cells, an increase in neutrophil count, an elevation of acute phase reactants, and a decrease in the monocyte count [25]. Both drugs have been shown to have anti-inflammatory properties, by reducing cytokine production, which would slow down the cytokine storm that deteriorates the condition of COVID-19 patients [5,31]. This would explain the significant increase in lymphocytes and the significant decrease in CRP that our patients experienced during their hospital stay. In addition, two well-recognized complications of COVID-19 are disseminated intravascular coagulation and macrovascular thrombosis with clear alterations in D-dimer and fibrinogen [32,33].

We found a significant decrease in fibrinogen levels in our patients during their hospital stay, which could be attributed to the modulating activity that nitazoxanide has on protein disulfide isomerase (PDI) given its involvement in the formation of atherosclerotic plaques and coagulation [5]. Regarding the neutrophil and monocyte count and the levels of ferritin and D-dimer, they followed the course of the disease without showing a significant change with the treatment. However, it has been shown that nitazoxanide has a significant role in reducing certain biochemical markers, among which the leukocyte count and the levels of LDH and D-dimer stand out, these being even significant in a meta-analysis [19].

Regarding the variable of mechanical ventilation requirement in the hospitalized patients’ group, we observed a lower proportion of patients (15.6%) when compared to that reported (20%) in a study carried out in a tertiary care center in the same city as our study [34]. A recent meta-analysis that included 33 randomized controlled trials, with a total of 10,489 COVID-19 patients treated with ivermectin, found a significant reduction in mechanical ventilation requirement (RR: 0.67, 95% CI: 0.47-0.96) compared with controls [16]. On the other hand, a meta-analysis of six randomized controlled trials with 1,412 COVID-19 patients treated with nitazoxanide showed a reduction in oxygen requirements (RR: 0.48, 95% CI: 0.39-0.59) compared with controls [17]. In our study, the median duration of hospitalization was nine days.

Interestingly, Ortiz-Brizuela et al. determined that in their center the median length of hospital stay was only five days [34]. However, controlled clinical trials comparing ivermectin with a placebo in hospitalized patients found the drug offered no benefit in terms of the length of hospital stay (seven days vs. seven days in the USA, and 9.6 days vs. 9.7 days in Bangladesh) [35,36]. In contrast, a meta-analysis involving nine studies found a significant reduction in time to clinical recovery in COVID-19 patients (MD: -1.21, 95% CI: -1.37 - -1.05, p<0.001) treated with ivermectin [37]. The median reported duration of hospitalization ranged widely (from 5 to 29 days), possibly due to the multiple factors involved in the discharge. One of these factors, already described, is the duration of the stay of patients who do not survive, which is generally shorter than those who do survive until discharge, with medians of 4-21 days and 4-53 days, respectively [37].

Finally, in the inpatient group, we found a 5% fatality rate. This rate is lower than that reported in the above-mentioned study by Ortiz-Brizuela et al. for hospitalized patients, which was found to be 7.1% [34]. Also, it is lower than the general fatality rate for confirmed COVID-19 patients in Mexico, which has been reported to range between 7.07% and 10.02% [38,39]. Furthermore, in a controlled clinical trial conducted in the USA involving hospitalized patients treated with ivermectin versus placebo, a fatality rate of 12.4% vs. 25.8%, respectively, was observed [13]. Most of the published meta-analyses have found a significant reduction in mortality in COVID-19 patients treated with ivermectin, mortality being the variable the drug impacts the most [13,15,40,41]. To the best of our knowledge, studies with nitazoxanide that analyze these variables have not been published.

Our study had a few limitations concerning both study groups. In the outpatient group, we did not include a placebo group or, where appropriate, the current standard of care, apart from the fact that a randomized and blinded study was not conducted. In addition, there was a loss of patients during their appointments a week and two weeks after starting their treatment, precluding the performance of their RT-PCR test. Regarding the group of hospitalized patients, the most important limitation, as in the other group, was the lack of inclusion of a comparison group with a standard of treatment.

Another limitation was that the laboratory studies were carried out on demand and did not follow a protocol to observe the evolution of the study variables more accurately over time. Moreover, we did not test the levels of pro-inflammatory cytokines involved in the pathophysiology of this disease. One more limitation in this group was the administration of other drugs in some patients, such as ruxolitinib and tocilizumab, which might interfere with the results. Tocilizumab has already been approved for use in certain hospitalized adult COVID-19 patients and ruxolitinib has shown benefits in certain clinical and prognostic parameters in these patients [42,43]. Finally, a limitation that affected both cohorts was the fact that some of the patients had already started with one or both studied drugs even before their evaluation, which could also influence the results.

## Conclusions

Despite the limitations of our study, our findings show that ivermectin and nitazoxanide could be useful in reducing the viral load, the requirement for mechanical ventilation, some proinflammatory and procoagulant parameters, and the fatality rate in COVID-19 patients. In addition, it is important to emphasize that no serious adverse effects were observed and that it is more likely that the high percentage of abdominal pain reported is within the drugs’ expected adverse event profile and not associated with the disease itself. Further research involving randomized controlled clinical trials evaluating this combination should be conducted to determine its true usefulness and safety profile.
